# STAT6 mutations compensate for CREBBP mutations and hyperactivate IL4/STAT6/RRAGD/mTOR signaling in follicular lymphoma

**DOI:** 10.1038/s41375-025-02525-6

**Published:** 2025-02-05

**Authors:** Qiangqiang Shao, Karan Bedi, Isabella A. Malek, Kerby Shedden, Sami N. Malek

**Affiliations:** 1 Departments of Internal Medicine, Division of Hematology and Oncology, University of Michigan, Ann Arbor, MI USA; 2 Biostatistics, University of Michigan, Ann Arbor, MI USA; 3 Statistics, University of Michigan, Ann Arbor, MI USA

**Keywords:** Lymphoma, Cell signalling

## Abstract

Activating mutations in *STAT6* are common in Follicular Lymphoma (FL) and transformed FL and various other B cell lymphomas. Here, we report RNA-seq based gene expression data on normal human lymph node derived B lymphocytes (NBC; *N* = 6), and primary human FL WT (*N* = 11) or mutant (*N* = 4) for *STAT6* before and after ex vivo stimulation with IL4. We found that STAT6 mutants result in broad based augmentation of IL4-induced gene expression. Unexpectedly, in FL with WT *STAT6* we measured reduced baseline and IL4-induced gene expression levels when compared with NBC lymphocytes or FL with *STAT6* mutations. We tracked the attenuated IL4/JAK/STAT6 response to co-existing *CREBBP* mutations and experimentally verified that intact *CREBBP* is required for the induction of many IL4-induced genes. One of the IL4-induced genes here identified is *RRAGD*, a small G-protein involved in lysosomal mTOR activation. We show that IL4 treatment induced RRAGD expression, that RRAGD is required for mTOR activation in lymphoma cells and that IL4-enhanced BCR signaling induced mTOR activation. The IL4 and BCR-induced mTOR activation was reduced by *CREBBP* mutants and augmented by mutant STAT6, establishing a link between STAT6 mutations and mTOR regulated pro-growth pathways in lymphoma.

## Introduction

Follicular lymphoma (FL) is the most common indolent B-cell lymphoma, with an incidence and prevalence of ~14,000 and ~100,000 cases, respectively, in the US [1]. FL remains incurable with conventional therapies, and the development of targeted FL-directed drugs lags behind the successes achieved in other lymphoid neoplasms [2–7].

FL has a varied clinical course and heterogeneous biology that is influenced by FL tumor cell-intrinsic factors and the microenvironment [2, 5, 6, 8–12]. FL cell-extrinsic factors, including FL B-cell to T cell- and macrophage interactions in the lymph node environment, as well as cytokine to FL cell interactions referred to as the microenvironment influence FL biology and outcome [13]. The 4/IL-4R ligand-receptor pair, which signals through Janus activated kinases (JAKs) and Signal transducer and activator of transcription 6 (STAT6) [14–18] has effects on FL B-cells and normal B-cells. IL-4 levels are elevated in the FL B cell microenvironment due to increased secretion by follicular helper T-cells (Tfh cells) [19–21].

We have previously reported on common activating hotspot mutations in STAT6 in follicular lymphoma and have demonstrated that selected IL4 responsive genes were expressed at higher levels at baseline and after IL4 treatment in FL carrying mutant *STAT6* as compared with FL with WT *STAT6* [22]. Prognostically, the presence of STAT6 mutations in FL as part of a gene mutation signature associated with shorter progression-free survival [23]. Other investigators have identified *STAT6* mutations in various lymphoid neoplasms, overall supporting a major role for *STAT6* mutations in B cell lymphoma biology and pathogenesis [24–27]. Since these initial reports, limited information has accrued as to the transcriptional program induced by IL4 and STAT6 in primary human B lymphocytes and the effects of mutant STAT6 proteins on this response [28]. Further, the biological properties imparted by mutant STAT6 on afflicted lymphoma cells and the lymphomatous LN microenvironment are also largely unknown.

For this study, we have measured gene expression in human lymph node derived normal B lymphocytes and in primary human FL B lymphocytes carrying WT or mutated *STAT6* at baseline and after IL4 stimulation. These unique and novel datasets allowed us to define the IL4- regulated transcriptome in human normal B lymphocytes and in primary FL. We show that STAT6 mutations augment IL4-induced gene expression for many IL4 responsive genes confirming an activating gain-of-function phenotype. We identify multiple novel IL4 induced genes and lay the foundation for biological follow-up studies guided by the known or anticipated function of these genes. While it is commonly believed that the IL4/JAK/STAT6 axis is activated not only in FL with STAT6 mutations but in most FL, we show that this axis is in fact attenuated in FL with WT STAT6 because of co-existing *CREBBP* mutations. One of the identified novel functions of *STAT6* mutations in FL is to restore or rescue the output of the IL4/JAK/STAT6 axis to normal or supranormal levels.

Focusing on one IL4 induced gene, *RRAGD*, a known lysosomal-resident mTOR regulator, we discover that IL4 induces RRAGD expression, which in turn augments BCR signaling induced mTOR activation. We proceed to show that STAT6 mutations hyperactivate IL4 and BCR induced mTOR activation thus identifying a novel mechanism of mTOR activation in lymphoma. This transcriptional RRAGD-mediated mechanism complements the mTOR activation that was previously reported to occur with FL-associated *RRAGC* or *ATP6V1B2* mutations thus broadening the importance of mutational control of mTOR signaling in FL [29–31].

In aggregate, these data provide novel insights into the target genes and biological effects of FL-associated STAT6 mutations with broader implications for other lymphoproliferative diseases, including PMBCL, DLBCL, HL, and t-FL, carrying such mutations and implications for novel therapy developments targeting the activated IL-4/JAK/STAT6/ RRAGD/mTOR axis in FL.

## Methods

### Ethics approval and consent to participate

Lymphoma patients were enrolled into two lymphoma repositories at the University of Michigan Rogel Comprehensive Cancer Center (IRBMED #HUM00007985 and IRBMED #HUM00017055) after signing informed consent documents approved by the University of Michigan Institutional Review Board (IRBMED). Genomic research on all specimens was approved through IRBMED #HUM00005467. All investigations were performed in accordance with ethical guidelines outlined in the declaration of Helsinki.

### Gene expression analyses of purified non-malignant human B lymphocytes isolated from lymph nodes and follicular lymphoma B lymphocytes using bulk RNA sequencing

Cryopreserved single-cell suspensions from human non-malignant lymph node (LN) biopsies or lymphomatous LNs were thawed, washed, and depleted of CD3^+^ T cells and CD14^+^ macrophages using Miltenyi beads and columns (Miltenyi, #130-050-101 and #130-050-201) following our published protocols [31]. The B lymphocytes were cultured for 4 h in the presence or absence of 10 ng/ml of IL4. Total RNA was purified using the Trizol reagent followed by RNAeasy column purification (Qiagen). RNA was assessed using the Bioanalyzer (Agilent #5067-1513) and a Qubit RNA High Sensitivity assay (Thermofisher # Q32855), and 2 ng of RNA input was processed for sequencing using the Takara SMART stranded kit (#634444).

The libraries were barcoded, pooled, and sequenced using paired-end, 151 bp sequencing at the University of Michigan Advanced Genomics Core. The reads were trimmed using Cutadapt v2.3 [32]. FastQC v0.11.8 was used to ensure the quality of data [33]. Fastq Screen v0.13.0 was used to screen for various types of contamination [34]. Reads were mapped to the reference genome GRCh38 (ENSEMBL), using STAR v2.7.8a [35] and assigned count estimates to genes with RSEM v1.3.3 [36]. Alignment options followed ENCODE standards for RNA-seq. The counts and TPMs for the technical replicates per treatment (control and IL4) were averaged for downstream analysis. FastQC was used in an additional post-alignment step to ensure that only high-quality data were used for expression quantitation and differential expression. Multiqc v1.7 compiled the results from several of these tools and provided a detailed and comprehensive quality control report [37]. Gene read counts calculated using RSEM were used to evaluate differential expression using DESeq2 v1.30.1 [38]. The sequencing files have been uploaded in the Gene Expression Omnibus (GEO) under accession # GSE261465.

### Antibodies, reagents, and immunoblotting

The following antibodies were used: Anti-STAT6 (Cell Signaling Technologies (CST), Danvers, MA, USA # 5397S), anti-phospho-STAT6 (CST # 9361S), anti-HA (CST 3724S), anti-CBP (CST, # 7389), anti-RagD (CST, # 4470), anti-HSP90 alpha/beta (Santa Cruz Biotechnology, Clone F-8, cat# sc-13119), anti-phospho-p70 S6 Kinase (Thr389) antibody (CST, Clone 1A5, # 9206), anti-β-Actin (CST, # 4967), anti-4E-BP1 (CST, Clone 53H11, # 9644), anti-phospho-4E-BP1 (Thr37/46) (CST, Clone 236B4, # 2855). The secondary antibodies used included anti-mouse IgG, HRP-linked (CST, 7076) and anti-rabbit IgG (CST, 7076), HRP-linked. A full listing of all reagents is provided in Supplementary Table 1.

### Generation of lymphoma cell lines expressing HA-tagged WT or mutated STAT6

Lentiviral transduction of HA-tagged WT or MUT STAT6 cDNA/ORF was performed via spinoculation of 2 × 10^6^ cells at 2600 rpm at 30 °C in the presence of polybrene at 4 µg/ml. The transduced cells were sorted on GFP brightness and expanded via culture. Expression of HA-STAT6 was confirmed via immunoblotting for HA and STAT6 and quantitatively compared with the expression of endogenous STAT6 via densitometry.

### Generation of *CREBBP* and *RRAGD* knock out lymphoma cell lines using CRISPR-Cas9

The targeting of *CREBBP, RRAGD* or *AAVS* in lymphoma cell lines was done using the pLENTI-CRISPRv.2 lentiviral vector and four guides each selected from the Brunello library [39]. Lentiviral production and cell infection was done as described [40]. Successful targeting of *CREBBP* or *RRAGD* was verified via immunoblotting of cell lysates made from targeted pools and these pools were used for the experiments described herein. The generation of bulk RNA sequencing data followed the methods detailed above. Cell line authentication was performed using the STR service at the university of Illinois tep_service@illinois.edu.

### IL4 treatment and BCR signaling studies in recombinant lymphoma cell lines

Recombinant HA-STAT6 WT or MUT expressing lymphoma cell lines or cell lines with disruptions of either *CREBBP*, *RRAGD*, or AAVS were cultured at 10^6^ cells per ml at 37 °C in 5% CO_2_ and ambient O_2_ in 10% FBS supplemented RPMI1640 medium or IMDM medium and were left untreated or treated with IL4 at 10 ng/ml for 4 h followed by BCR crosslinking with goat F(ab’)2 anti-human IgM (Southern Biotech, #2022-01) or goat F(ab’)2 anti-human IgG (Southern Biotech, #2042-01) at a concentration of 10 μg/ml for 10’. After stimulation, the cells were pelleted and lysed using a lysis buffer containing 1% NP-40 detergent (Dot Scientific, #DSC41010), 150 mM NaCl (Dot Scientific, #DSS23020), 25 mM Tris pH 8.0 (Sigma, #T6066), 20 mM NaF (Fisher, #S299), 2 mM EGTA (Sigma, #E3889), 2 mM EDTA (Sigma, #ED2SS), and supplemented with protease inhibitors (Sigma, #P8340), phosphatase inhibitors (Sigma, #P0044), sodium orthovanadate (Sigma, #450243), and PMSF (Thermo Fisher Scientific, #36978). The detergent-soluble fraction of the cell lysates was obtained by centrifugation at 14,000 rpm for 10’. The protein samples were fractionated by SDS-PAGE and prepared for immunoblotting using standard protocols. Cell lysates from parental 293 T cells or transfected with a RRAGD cDNA (Addgene, # 19316) and Hela cells treated with and without insulin were used as blotting and epitope controls.

### Purification, culture and IL4 and anti-IgM treatment of human lymphnode derived non-malignant B lymphocytes

Cryopreserved single-cell suspensions derived from human lymph node (LN) biopsy samples were thawed and cultured at 37 °C with 5% CO_2_ using dedicated B cell growth media (ImmunoCult™ Human B Cell Expansion Kit; Stemcell Technologies, #100-0645). To purify and enrich B lymphocytes, cells were washed and resuspended in a degassed BSA/EDTA buffer (1X PBS, 0.5% BSA, and 1 mmol/L EDTA). The cell suspension was supplemented with CD3 MicroBeads (human, Miltenyi Biotec, #130-050-101) and CD14 MicroBeads (human, Miltenyi Biotec, #130-050-201) and purified over LS columns (Miltenyi Biotec, #130-042-401) using a QuadroMACS Separator (Miltenyi Biotec, #130-091-051). Purified B lymphocytes were then briefly expanded and treated with either vehicle or 10 ng/ml IL4 for 6 h. An aliquot of the purified cells was stimulated with Goat F(ab’)2 Anti-Human IgM (Southern Biotech, #2022-01) at a concentration of 10 μg/ml for 10’. Cells were pelleted and lysed using the lysis buffer conditions as above and analyzed by immunoblotting using indicated antibodies.

### Statistical methods

Statistical analyses were conducted using GraphPad Prism (Version 9.1.2 for Windows 64-bit) unless specified otherwise. Statistical significance was assessed at a 5% significance level. *P*-value calculations were two-tailed unless specified otherwise. The Mann–Whitney or Wilcoxon test (unpaired, non-parametric) was applied to compare variables between two groups unless stated otherwise. For multiple group comparisons, ANOVA or Freidman tests with appropriate post hoc comparisons were conducted. The Brown-Forsythe test was performed for each data set to assess equality of variance between groups as well. All statistical methods for each panel were specified in the corresponding figure legends. Statistical results were presented as follows: ns (non-significant), *p < 0.05, **p < 0.01, ***p < 0.001. All collected data were used for analysis without intentional exclusion by any criteria. The number of biological and technical replicates for individual experiments, as well as additional detailed information for each statistical test performed, is listed in Supplementary Table 2.

#### Data sharing and management plan

All constructs and cell lines will be made available upon request. The normal B cell and FL RNA seq sequencing files have been uploaded in the Gene Expression Omnibus (GEO) under accession # GSE261465. The bulk RNA seq lymphoma cell line sequencing files have been uploaded in GEO under accession # GSE280295.

#### Additional methods

All experiments were performed using internationally accepted laboratory standards. Recombinant DNA work was approved under the institutional biosafety committee approval # IBCA00001121_AR04. A listing of all reagents used is provided in Supplementary Table 1.

## Results

### The IL4 responsive transcriptome in primary human lymph node derived normal B lymphocytes and primary human Follicular Lymphoma B lymphocytes WT or mutated for *STAT6*

To identify the gene expression changes induced by IL4 in primary human LN derived normal B lymphocytes and primary human Follicular Lymphoma B lymphocytes, we performed RNA-seq on ribosomal RNA depleted total RNA isolated from purified (via depletion of T cells and macrophages) human LN derived normal B lymphocytes (NBC; *N* = 6), human primary FL WT for *STAT6* (*N* = 11) and primary FL MUT for *STAT6* (*N* = 4) before and after ex vivo stimulation with IL4 at 10 ng/ml for 4 h. The resulting sequence data were quality controlled and gene expression normalized and compared using standard approaches (*see methods;* Supplementary Table 3). PCA analyses demonstrated that samples grouped by sample ID with minor effects of IL4 treatment and that NBC cells separated from FL samples as expected (Supplementary Fig. 1).

**Fig. 1:**
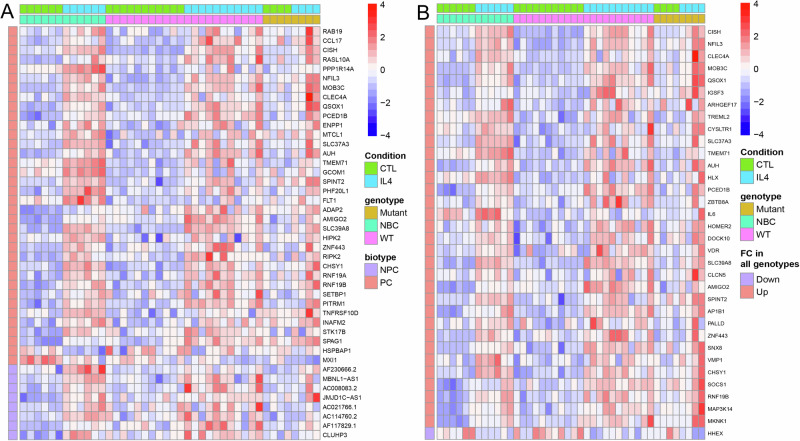
Identification of IL4 regulated genes in normal human lymph node derived B cells, FL B cells with WT STAT6 and FL B cells with MUT STAT6 (RNAseq results; heatmaps). **A** Heatmap displays genes that are ≥1.5-fold upregulated or ≥1.5-fold downregulated in the normal human LN derived B cells (NBC), sorted by fold-change, with a mean TPM value ≥ 1 (for IL4 treated replicates in upregulated) or ≥1 (for CTL replicates in downregulated), pAdj (q) ≤ 0.1 and compared to the other sets. NBC *N* = 6, FL B cells with WT STAT6 *N* = 11; FL B cells with MUT STAT6 *N* = 4. Heatmap shows row wise scaled normalized counts. **B** Heatmap (core IL4-induced gene program) shows genes that are ≥2-fold upregulated or ≥2-fold downregulated in the NBC, FL B cells with WT STAT6 and FL B cells with MUT STAT6. Heatmap shows row wise scaled normalized counts. STAT6 Signal Transducer and Activator of Transcription 6, IL4 interleukin 4, IL4 interleukin 4, FL follicular lymphoma, RNAseq ribonucleic acid sequencing, WT wild type, MUT mutated, NBC normal B cells.

Next, we performed various pairwise comparisons (e.g., NBC lymphocytes ± IL4 treatment or primary FL WT or MUT for STAT6 ± IL4 treatment) of gene expression in selected groups. The genes with >1.5-fold induction (*q* < 0.1) or >1.5-fold suppression in NBC lymphocytes are displayed in the RNA seq based scaled (scaled = [transcripts per million (TPM) values minus TPM row mean] divided by the standard deviation) heatmap for these genes in all three data sets (NBC, FL WT or MUT for STAT6) in Fig. 1A. In the NBC lymphocyte data, we identified 44 genes (protein coding and other biotypes, including lncRNAs) with a significant change in expression following IL4 treatment (Fig. 1A; Supplementary Table 4). A subset of the strongly induced protein coding genes included (all log_2_-fold induction): *RAB19* (4 x), *CCL17* (3.9 x), *CISH* (3.7 x), *RASL10A* (3.3 x), *PPP1R14A* (3.1 x), *NFIL3* (3 x), *MOB3C* (3 x), *CLEC4A* (2.7 x), *QSOX* (2.5 x), *PCED1B* (2.4 x), *AUH* (2.1 x), *SLC37A3* (2.3 x) and of interest the mTOR regulator *RRAGD* (1.2 x). We also identified 34 genes that were >2-fold induced in all three data sets and displayed the scaled RNA seq based TPM values for upregulated protein coding genes in the heatmap in Fig. 1B.

**Fig. 2:**
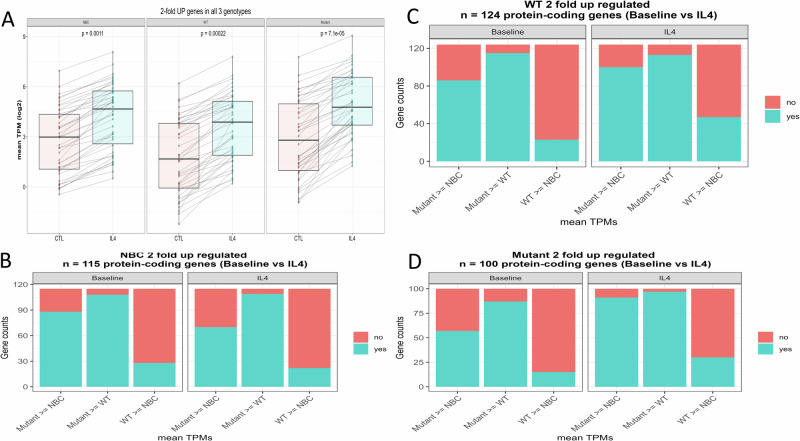
Elevated baseline and IL4-induced expression of the majority of IL4 inducible genes in normal human lymph node derived B cells (NBC) or FL B cells carrying MUT STAT6 when compared with the expression in FL B cells carrying WT STAT6. **A** Baseline or CTL: no IL4 treatment, IL4: ex vivo IL4 treatment. Box plots. Y-axis: mean TPM on a log2-scale. Each dot and line represent one gene. *P*-values computed via Wilcox test. **B–D** Stacked histograms: In each stacked histogram the fraction of genes expressed higher in the indicated sample set is colored in blue. IL4 inducible genes were defined with a fold change ≥2-fold in each of the three data sets independently (NBC: normal B cells, WT: FL B STAT6 WT, Mutant: FL B STAT6 MUT). STAT6 Signal Transducer and Activator of Transcription 6, IL4 interleukin 4, FL follicular lymphoma, CTL control, log logarithmic scale, WT wild type, MUT mutated, NBC normal B cells, TPM transcripts per million.

In previous work, we showed that MUT STAT6 increases the affinity for STAT6 binding motif containing DNA oligonucleotides and augmented the expression of STAT6 signature target genes (*CISH, FCER2, NFIL3*) [22]. We compared the magnitude of IL4-induced gene expression changes in FL samples with WT or MUT STAT6. We found a substantial increase in the baseline expression and IL4 stimulated expression in most genes in the MUT *STAT6* FL samples when compared with the WT samples, confirming a strong genome-wide gain-of-function phenotype for the STAT6 MUTs (Fig. 2A–D).

We measured the expression of selected genes (*CCL17, QSOX1, MOB3C* and *CLEC4A*) by qRT-PCR in mRNA/cDNA isolated from normal B lymphocytes and FL with WT or MUT STAT6 in an independently isolated and IL4 treated set of biological samples, which included samples not contained in the RNAseq data. As can be seen in (Supplementary Fig. 2, upper panels), the baseline and IL4-induced expression was higher in the FL STAT6 MUT samples compared with FL STAT6 WT samples. Similar findings were apparent comparing gene expression in NBC compared with FL WT STAT6 samples.

Amongst the group of IL4-induced genes in the FL STAT6 MUT group, we identified genes that were not induced in the FL STAT6 WT or NBC samples, indicating a neomorphic gain-of-function of the STAT6 mutant proteins. Amongst these genes, we highlight the NOTCH ligand *JAG2*, the atypical homeodomain protein *BARX2*, and *CHM2*, a GAP for p21Rac, all of which are likely relevant to lymphoma pathogenesis and the subject of our ongoing studies. We confirmed the STAT6 MUT specific expression of BARX2, JAG1 and CHM2 via qRT-PCR as above (Supplementary Fig. 2 lower panels).

While many of the IL4-induced genes have interesting biological functions with relevance to lymphomagenesis, we became interested in RRAGD, a G-protein known to participate in lysosomal mTOR activation and a binding partner of RRAGA or RRAGB as part of the LAMPTOR/ RRAGA, B, C and D/ FLCN/ mTOR activation complex [41, 42]. We selected RRAGD for biological follow-up studies as detailed further below.

### The IL4/JAK/STAT6 responsive transcriptional program is attenuated in *CREBBP* mutated FL carrying WT *STAT6* and restored by *STAT6* mutations

We compared the baseline- and the IL4-induced gene expression levels in normal (NBC) versus malignant B lymphocytes and in FL B lymphocytes carrying WT or MUT *STAT6*. Unexpectedly, we detected substantially lower baseline and IL4-induced gene expression for the majority of genes in FL cells carrying *STAT6* WT when compared with NBC lymphocytes (*p* = 0.04) or with FL B lymphocytes with MUT *STAT6* (*p* = 0.04), indicative of a downregulation of the IL4/JAK/STAT6 signaling axis in FL carrying *STAT6* WT (Fig. 2A–D). FL are characterized by a high frequency of mutations in *CREBBP* and *KMT2D/MLL2* [43, 44]. We and others have previously reported on a strong co-occurrence of mutations in *STAT6* and *CREBBP* in FL implying a functional interaction [22, 45, 46]. Of the eleven FL cases with WT STAT6 subjected to RNA-seq, nine (82%) carried *CREBBP* mutations, while all four STAT6 MUT FL were *CREBBP* mutated. We surmised that *CREBBP* mutations attenuated the IL4/STAT6 signaling pathway in FL likely at the level of impaired gene transactivation and that STAT6 mutants compensated for this defect [47].

### Mutations in *CREBBP* in FL attenuate the IL4/JAK/STAT6 responsive transcriptional gene expression program

**Fig. 3:**
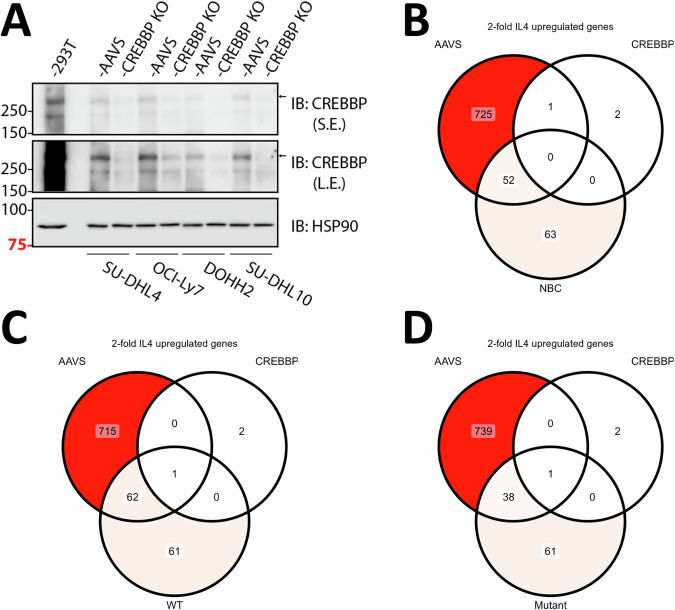
CREBBP is required for the induction of IL4 inducible genes. **A** The *CREBBP* gene or AAVS (control) was targeted with pooled CRISPR-Cas9 guide pools in four lymphoma cell lines (SU-DHL4, OCI-LY7, SU-DHL10, DOHH-2) and the pools were analyzed for CREBBP protein expression by immunoblotting (S.E. short exposure; L.E. long exposure). Total ribo depleted RNA from cells pools that were untreated or ex vivo IL4 treated were subjected to RNA-seq. Venn diagrams show the number of genes that are >2-fold upregulated by IL4 in AAVS targeted cell line pools (controls), *CREBBP* targeted cell line pools (*CREBBP −/−)*, and in normal human B cells (**B**), or FL B cells with WT STAT6 (**C**) or FL B cells with Mutant STAT6 (**D**). IL4 interleukin 4, CREBBP CREB binding protein, AAVS adeno-associated virus integration site, qRT-PCR quantitative reverse transcriptase polymerase chain reaction, FL follicular lymphoma, WT wild type, MUT mutated.

To test the hypothesis that *CREBBP* status influenced STAT6-mediated gene induction, we created isogenic lymphoma cells lines (OCI-LY7, SUDHL4, SUDHL10, DOHH2) via CRISPR-Cas9 mediated disruption of *CREBBP* or adenovirus associated integration site (AAVS; control). We confirmed loss of CREBBP expression in the targeted cell line pools via immunoblotting (Fig. 3A). We treated the cell lines with IL4 for 4 h or left them untreated and performed bulk RNAseq on ribo RNA depleted purified RNA. We reviewed the expression of known IL4 inducible genes as defined by the RNA seq results from NL and FL B lymphocytes detailed above in these cell lines. Overall, the loss of CREBBP ablated the IL4 inducible gene expression program in these cell lines, confirming that CREBBP is a major positive cofactor for IL4-induced gene expression (Fig. 3B–D and Supplementary Table 5). This novel finding likely explains the lowered baseline and IL4-induced gene expression in FL carrying WT *STAT6* and MUT *CREBBP* [47]. Furthermore, the data imply that FL selects for *STAT6* mutations to compensate for the attenuation of the IL4/JAK/STAT6 axis in FL by mutant *CREBBP*.

### Expression of STAT6 mutants in *CREBBP* −/− cells rescues IL4-induced gene expression

**Fig. 4:**
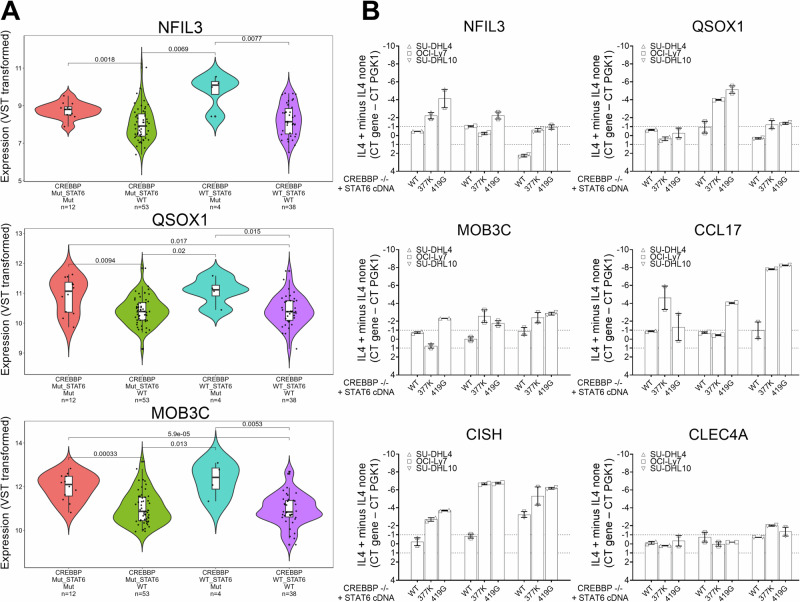
STAT6 mutations rescue the expression of IL4 inducible genes in CREBBP −/− lymphoma cells. **A** RNA seq based expression of selected IL4 responsive genes grouped by four FL genotypes (*CREBBP* and *STAT6*) based on the data published by Dreval et al., *Blood* [48]. Please also see Supplementary Fig. 3. **B** The expression of six IL4 inducible genes in three CREBBP −/− lymphoma cell lines expressing lentivirally transduced HA-tagged STAT6 WT or STAT MUT (p.377 K and p.419 G). Displayed is the difference of delta Ct values (CT mean gene – Ct mean PGK1) from samples with or without IL4 stimulation. STAT6 Signal Transducer and Activator of Transcription 6, IL4 interleukin 4, FL follicular lymphoma, Ct cycle threshold.

We plotted the expression of the IL4 inducible genes shown in Fig. 1B by genotype (*CREBBP* WT or MUT and *STAT6* WT or MUT) using the dataset published by Dreval et al.*, Blood* [48]. We found that the majority of genes were expressed at higher levels in *CREBBP* MUT/*STAT6* MUT FL when compared with *CREBBP* MUT/*STAT6* WT FL (Fig. 4A, Supplementary Fig. 3 and Supplementary Table 6).

We transduced the *AAVS* and *CREBBP* −/− targeted cell lines SU-DHL4, OCI-LY7 and SU-DHL10 with FG9 lentivirus containing STAT6 WT or MUT cDNAs (p.377 K and p.419 G) followed by cell sorting on GFP, IL4 treatment of GFP+ cell pools, RNA isolation and qRT-PCR for the IL4 responsive genes *NFIL3, QSOX1, MOB3C, CCL17, CISH* and *CLEC4*. We found that STAT6 mutant expression elevated the expression of most of these genes in the majority of the reconstituted cell lines (Fig. 4B) and only CLEC4 expression demonstrating no change.

Together these data demonstrates that *STAT6* MUT rescue the expression of IL4 inducible genes in FL with *CREBBP* mutations.

### IL4 upregulates the expression of the mTOR regulator RRAGD

**Fig. 5:**
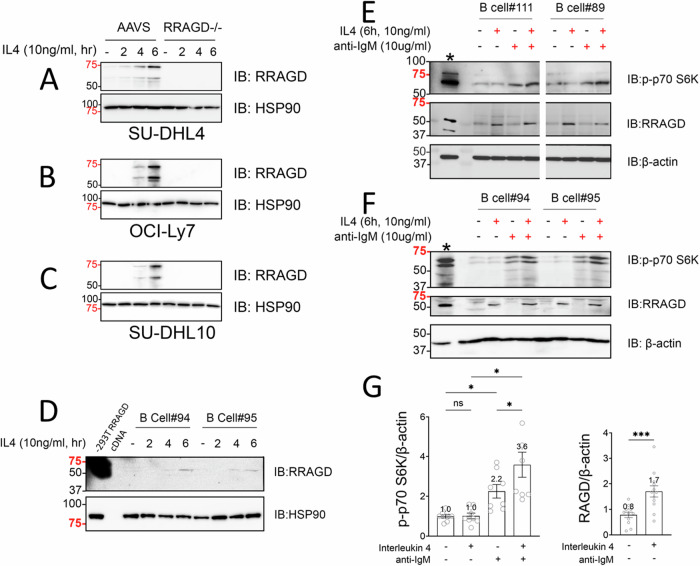
IL4 induces RRAGD expression and combined IL4 treatment and BCR crosslinking hyperactivates mTOR signaling. **A–C** Lymphoma cell line pools transduced with lentiviral CRISPR-Cas9 guide pools targeting AAVS (control) or *RRAGD*, were treated ex vivo with IL4 for 0–6 h (time course). Cell lysates were made and prepared for immunoblotting using the indicated epitopes. **D** Non-malignant normal human LN derived B cells were purified via Miltenyi columns and depletion of CD3 T cells. Purified B cells were treated ex vivo with IL4 for 0–6 h (time course). Cell lysates were made and prepared for immunoblotting using the indicated epitopes. **E**, **F** Non-malignant normal human LN derived B cells (*N* = 4) were purified via Miltenyi columns and depletion of CD3 T cells. Purified B cells were left untreated or treated ex vivo with IL4, anti-IgM or both as indicated. Cell lysates were prepared for immunoblotting using the indicated epitopes. **G** densitometry results for p-p70-S6K and RRAGD normalized to actin for all conditions for four primary human LN-derived B lymphocyte samples (# 111, 89, 94, and 95) combined. Left panel: One-way Anova with post hoc Tukey’s test. Right panel: paired nonparametric Wilcoxon test. *p < 0.05, ***p < 0.001. IL4 interleukin 4, BCR B cell receptor, AAVS adeno-associated virus integration site, CRISPR-Cas9 clustered regularly interspaced short palindromic repeats CRISPR associated protein 9, IgM immunoglobulin M, ANOVA analysis of variance.

Next, we followed up on our finding of transcriptional upregulation of RRAGD by IL4 with dedicated RRAGD immunoblotting kinetics in lymphoma cell lines treated for up to 6 h with 10 ng/ml of IL4. We found that IL4 treatment resulted in substantial induction of RRAGD protein expression (Fig. 5A–C) [49]. We performed experiments in purified non-malignant normal human lymph node derived B lymphocytes treated with IL4 and obtained similar results (Fig. 5D–F).

### IL4 and BCR signaling augments mTOR activation in human lymph node derived non-malignant B lymphocytes

IL4 is produced in lymph nodes by specialized T cells (Tfh cells) and thus available in the microenvironment of FL B lymphocytes [19]. We measured mTOR activation in purified non-malignant human lymph node derived B lymphocytes (*N* = 4) pre-treated for 4 h with IL4 and activated via sIgM (BCR) crosslinking. We found that IL4 treatment induced RRAGD 2.1-fold and augmented BCR induced mTOR activation as detected via elevated expression of p-S6K 3.6-fold (Fig. 5E–G).

### RRAGD is essential for mTOR signaling in lymphoma

**Fig. 6:**
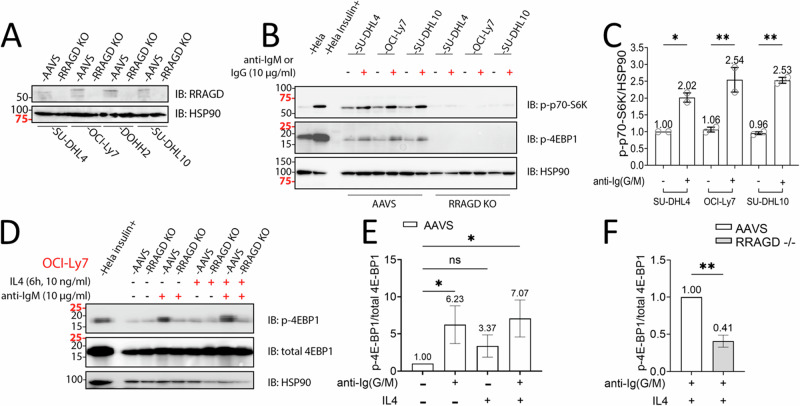
RRAGD is required for S6 Kinase phosphorylation in lymphoma. **A** The *RRAGD* gene or AAVS (control) was targeted with lentivirus carrying pooled CRISPR-Cas9 guides in four lymphoma cell lines and following puromycin selection the pools were analyzed for RRAGD protein expression by immunoblotting. **B**, **C** RRAGD −/− or AAVS targeted lymphoma cell line pools were treated with anti-IgM or anti-IgG for 10’, cell lysates were made, and protein prepared for immunoblotting using the indicated epitopes. Densitometry data for mean p-p70-S6K:HSP90 for AAVS control cells are shown, while signals for RRAGD −/− cells were close to background. One-Way ANOVA with post hoc Tukey’s test. Densitometry based on three cell lines and *N* = 2 repeats *p < 0.05, **p < 0.01. **D–F** RRAGD −/− or AAVS targeted lymphoma cell line pools were treated +/− IL4 for 6 h and +/− anti-IgM for 10’, cell lysates were made and protein prepared for immunoblotting using the indicated epitopes. **D** A representative blot for OCI-LY7. **E** Densitometry of mean p-4E-BP1:total 4E-BP1 for AAVS control cells +/− IL4 for 6 h and +/− anti-IgM/G. One-Way ANOVA with post hoc Dunn’s test. **F** Densitometry of comparative data for AAVS and RRAGD −/− cells; Mann–Whitney test. Densitometry based on three cell lines and *N* = 2 repeats. ns, not significant, *p < 0.05, **p < 0.01. AAVS adeno-associated virus integration site, CRISPR-Cas9 clustered regularly interspaced short palindromic repeats CRISPR associated protein 9, IgM immunoglobulin M, IgG immunoglobulin G, IL4 interleukin 4, ANOVA analysis of variance.

The RRAGD protein or the RRAGC protein, the latter being mutated and activated in 10% of FL [29, 30], form heterodimeric complexes with either RRAGA or RRAGB on the lysosomal surface [41, 42]. There have been reports of relative mTOR substrate selection/specificity imparted on mTOR by various RRAG heterodimers [50]. To better define the role of RRAGD in lymphoma cells, we generated *RRAGD* knock-out cell lines via CRISPR-Cas9 gene disruption and confirmed loss of RRAGD expression in resulting cell pools (Fig. 6A). Next, we treated these pools with anti-IgM or anti-IgG to activate BCR signaling and mTOR and measured phosphorylation of S6K and 4EF-BP1. We found that *RRAGD* disruption completely blocked S6K phosphorylation in four RRAGD −/− lymphoma cell lines (Fig. 6B, C), and substantially reduced phosphorylation of 4EF-BP1 (Fig. 6D–F), indicating a non-redundant requirement for RRAGD in mTOR activation in lymphoma, which was a surprising finding [50]. The data also indicate that RRAGC, which is expressed highly in these lines cannot compensate for the loss of RRAGD.

### Mutations in *CREBBP* attenuate IL4 and BCR induced mTOR signaling

To study the effects of CREBBP loss of function on IL4 and BCR activated mTOR signaling, we employed three CREBBP −/− or AAVS targeted cell lines (SUDHL4, SUDHL10 and OCI-LY7). The cell pools were treated with IL4 at 10 ng/ml for 6 h or not and either anti-IgM or anti-IgG or solvent for the last 10’. We made detergent protein lysates and prepared lysates for immunoblotting with indicated antibodies. Following IL4 and anti-Ig treatment, we detected strong up-regulation of p-S6K (Supplementary Fig. 4A, B) in control cells. The phosphorylation of S6K was substantially reduced in CREBBP −/− cells indicating that CREBBP loss impairs mTOR activation (Supplementary Fig. 4C, D).

### STAT6 mutations hyperactivate the IL4/JAK/STAT6/RRAGD/mTOR signaling axis

**Fig. 7:**
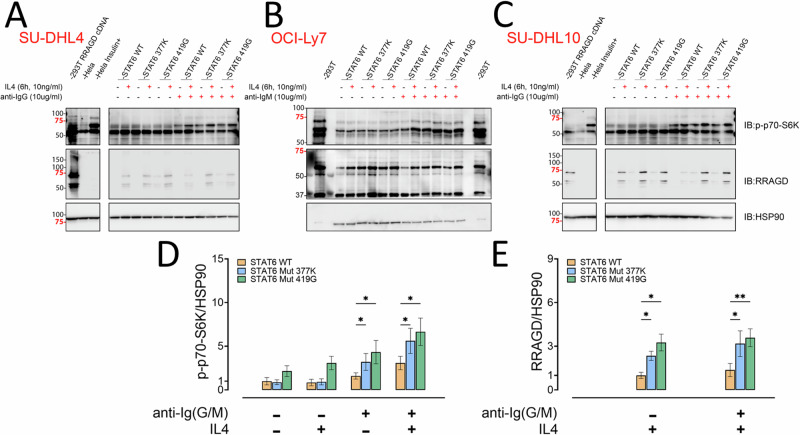
STAT6 mutations hyperinduce RRAGD expression and IL4 and BCR induced mTOR signaling. Three lymphoma cell lines were lentivirally transduced with cDNA/ORFs for WT or MUT STAT6 and cells sorted via GFP fluorescence. Cells were stimulated with IL4 and BCR crosslinking as indicated or left untreated and detergent lysates prepared for immunoblotting using the indicated epitopes. **A–C** Representative immunoblotting results for *N* = 2 independent experiments per cell pool. **D** Results from densitometry for phospho-S6K normalized to HSP90 for indicated conditions across three cell lines (*N* = 6). Two-way ANOVA with post hoc Holm Šidák test *p < 0.05. **E** Results from densitometry for RRAGD normalized to HSP90 for indicated conditions for *N* = 2 independent experiments across three cell lines (*N* = 6 measurements). Two-way ANOVA with post hoc Holm Šidák test *p < 0.05; **p < 0.01. STAT6 Signal Transducer and Activator of Transcription 6, IL4 interleukin 4, WT wild type, MUT mutated, GFP green fluorescence protein, cDNA complementary DNA, ORFs open reading frames, IL4 interleukin 4, BCR B cell receptor, ANOVA analysis of variance.

We generated recombinant lymphoma cell line pools (SUDHL4, OCI-LY7, SUDHL10) via lentiviral transduction of WT or MUT (p.377 K, p.419 G) STAT6 sorted to purity via FACS. We pre-treated these cell lines with IL4 for 6 h followed by sIgG or sIgM (BCR) crosslinking. We found that IL4 treatment augmented BCR induced mTOR activation, as detected via elevated expression of p-S6K, which was substantially elevated in the cell lines expressing MUT STAT6 (Fig. 7A–E). The combined treatment with IL4 and BCR crosslinking resulted in strong induction of RRAGD, which was substantially augmented by the STAT6 mutants.

Summarizing, STAT6 mutations in FL hyperactivate the IL4/STAT6/JAK/RRAGD/mTOR signaling axis thereby facilitating lymphoma cellular growth and metabolism.

## Discussion

Activating mutations in STAT6 are common in human lymphomas but details of their biological and functional consequences are sparse [22, 24, 26, 27, 46, 51]. Activating mutations in STAT6 also underly allergic hyperactivation syndromes in humans [52]. Here, we present the delineation of the IL4-induced transcriptome via RNAseq in normal human lymph node derived B lymphocytes and in FL B lymphocytes WT or mutant for STAT6. Data are unique as human samples to perform such studies are rare. Overall, we report on the novel findings that (i) STAT6 mutations are gain-of-function resulting in elevated gene induction amplitudes across most IL4 responsive genes, (ii) that multiple novel genes with diverse functions relevant to lymphoma cells are IL4-induced, providing a starting point for in-depth mechanistic dissection, (iii) that *CREBBP*, which is commonly mutated in FL and almost always mutated in *STAT6* mutated FL is a co-factor in STAT6 target gene induction and that FL with WT *STAT6* have an attenuated IL4/JAK/STAT6 induced gene expression program, (iv) that mutant STAT6 rescues the *CREBBP* mutation associated attenuation of the IL4/JAK/STAT6 induced gene expression program, (v) that the mTOR regulator RRAGD is critical for mTOR activation in lymphomas and is transcriptionally upregulated via IL4, (vi) that IL4 via RRAGD induction sensitizes the mTOR pathway to heightened activation via BCR crosslinking, and, vii) that STAT6 mutation strongly augment IL4 and BCR signaling activated mTOR signaling.

The identification of novel genes and their function that are induced by IL4 treatment in normal B lymphocytes and FL B lymphocytes will allow for a detailed dissection of the consequences of activation of the IL4/JAK/STAT6 axis and its upregulation by STAT6 mutations. The IL4/JAK/STAT6mut axis regulates a variety of pathways and processes, including T cell recruitment into the FL microenvironment via massive upregulation of the cytokine CCL17, lymphoma cell surface signaling via CLEC4, negative feedback regulation of JAK/STAT signaling via CISH and SOCS, redox sensing and protein disulfide formation via QSOX1, Tfh cell differentiation via IL6, circadian gene transcription, including IgE and apoptosis regulation via NFIL3, transcriptional regulation via AUH, HLX, HHEX and AHR, cytokine signaling via IL27RB, NFKB regulation via NFKBIZ and multiple other processes.

Is a broad-based upregulation of the amplitude of induction of IL4-induced genes the major mechanism of action of STAT6 mutants? The answer is yes, as the majority of upregulated genes in STAT6 mutated FL are also upregulated in normal B lymphocytes and FL with STAT6 WT status albeit mostly at lower levels. However, we detected a few genes, which in IL4 treated cells were specifically induced in STAT6 mutated FL, suggesting an acquired ability of STAT6 mutants to activate these genes. These genes, among others, include the NOTCH ligand JAG1, the atypical homeodomain protein BARX2 and CHN2 (chimerin), a GAP for p21Rac, all of which are likely relevant to lymphoma pathogenesis and the subject of our ongoing studies [53].

In summary, the data provided here provide insights in the mutational activation of the IL-4/JAK/STAT6/RRAGD/mTOR axis in FL and provide fertile grounds for future research, including but not limited to the exploration of the IL-4/JAK/STAT6/RRAGD/mTOR axis as a therapeutic target for small molecules (JAK inhibitors or mTOR inhibitors) in FL [54–56].

## Supplementary information

Supplementary legends and figures 1 to 4

Supplementary Table 1

Supplementary Table 2

Supplementary Table 3

Supplementary Table 4

Supplementary Table 5

Supplementary Table 6
