# Supplementary material for: STAT6 mutations compensate for CREBBP mutations and hyperactivate IL4/STAT6/RRAGD/mTOR signaling in follicular lymphoma
Source: Leukemia. 2025 Feb 5;39(4):899–908. doi: 10.1038/s41375-025-02525-6 (PMC11976298; doi:10.1038/s41375-025-02525-6)
Supplement: Supplementary file 1 — Supplementary legends and figures 1 to 4 [file 41375_2025_2525_MOESM1_ESM.pdf]

STAT6 mutations compensate for CREBBP mutations and hyperactivate IL4/STAT6/RRAGD/mTOR signaling in Follicular Lymphoma

Qiangqiang Shao[1], Karan Bedi[2], Isabella A. Malek[1], Kerby Shedden[3] and Sami N. Malek[1,#]

From the Departments of Internal Medicine[1], Division of Hematology and Oncology[1], Biostatistics[2], and Statistics[3], University of Michigan, Ann Arbor, MI, USA

Supplementary Figure and Table Legends

Figure S1: Principal component analyses (PCA) of RNA seq results for normal B cells (NBC N=6) and FL B cells with WT STAT6 (WT N=11) or mutated STAT6 (MUT N=4) per group and combined. IL4: samples treated *ex vivo* with IL4 prior to RNA extraction.

Figure S2: Results from qRT-PCR based expression analyses of IL4 inducible genes in mRNA/cDNA isolated from purified NBC, FL B cells with WT STAT6, or FL B cells with MUT STAT6. B-lymphocytes were purified from cryopreserved LN biopsies via column-based depletion of CD3+ T cells and CD14+ macrophages and cultured for 4 h +/- 10ng/ml of IL-4. Random primed cDNA was made from total RNA. Displayed are delta Ct values (mean Gene – mean PGK1) x (-1).

Figure S3: STAT6 mutations rescue the expression of IL4 inducible genes in CREBBP -/- lymphoma cells. RNA seq based expression of selected IL4 responsive

genes grouped by four FL genotypes (*CREBBP* WT or MUT and *STAT6* WT or MUT) based on the data published by Dreval et al, Blood [48].

Figure S4: Reduced mTOR activity in CREBBP -/- lymphoma cell lines. For CREBBP knock-down validation, please see Figure 3A. **A:** Representative immunoblotting results (N≥2 experiments on three cell lines) for detergent cell lysates made from three lymphoma cells line with CRISPR-Cas9 mediated disruption of *CREBBP* or *AAVS* +/- treatment with IL4 and +/- anti-IgM or anti-IgG. **B-D:** Densitometry results for indicated band intensity ratios under indicated experimental conditions. B: Two-way ANOVA with post hoc Šídák analysis, ns= not significant, * $p<0.05$. C-D: Wilcoxon test * $p<0.05$.

Supplementary Table S1: Reagent information.

Supplementary Table S2: Statistical methods used itemized by figures.

Supplementary Table S3: Normalized RNA seq based gene expression for NBC N=6, FL B cells with WT STAT6 N=11; FL B cells with MUT STAT6 N=4 +/- *ex vivo* IL4 stimulation. The sequencing files have been uploaded in the Gene Expression Omnibus (GEO) under accession # GSE261465.

Supplementary Table S4: Genes that are upregulated or down-regulated in NBC N=6, FL B cells with WT STAT6 N=11; FL B cells with MUT STAT6 N=4 +/- *ex vivo* IL4 stimulation. TPM values on a log2-scale.

Supplementary Table S5: Normalized RNA seq based gene expression for four lymphoma cell lines pools with *CREBBP* -/- or *AAVS* (control locus) CRISPR Cas9 targeting +/- *ex vivo* IL4 stimulation. The bulk RNA seq lymphoma cell line sequencing files have been uploaded in GEO under accession # GSE280295.

Supplementary Table S6: Expression of IL4 inducible genes by *CREBBP* and *STAT6* mutation status in the dataset published by Dreval et al, *Blood* [48].

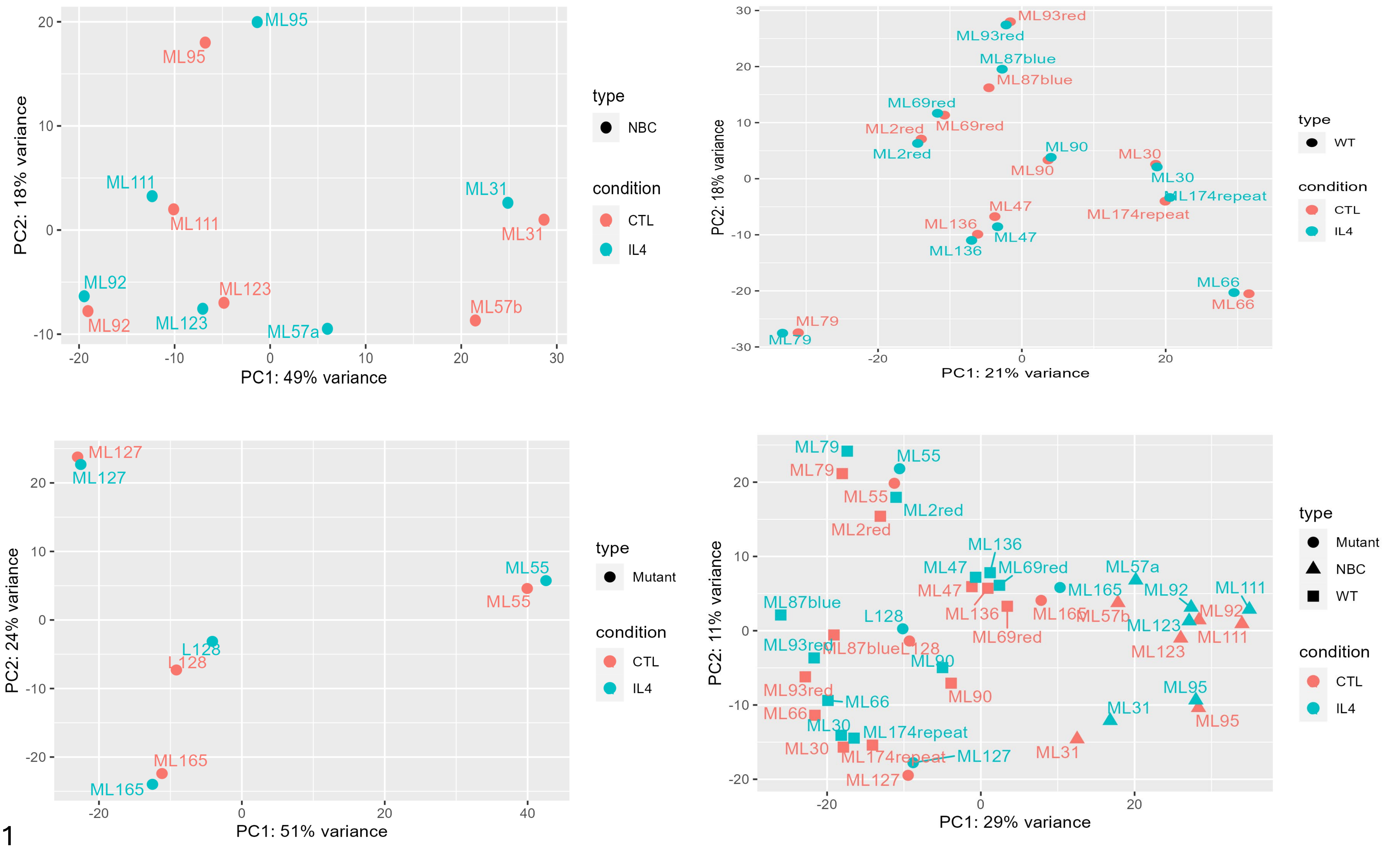

PC1: 49% variance
PC2: 18% variance
type
NBC
condition
CTL
IL4
ML95
ML111
ML31
ML92
ML123
ML57a
ML57b
PC1: 21% variance
PC2: 18% variance
WT
ML93red
ML87blue
ML69red
ML2red
ML90
ML30
ML174repeat
ML47
ML136
ML66
ML79
PC1: 51% variance
PC2: 24% variance
Mutant
ML127
ML55
L128
ML165
PC1: 29% variance
PC2: 11% variance

Figure S1

Figure S2

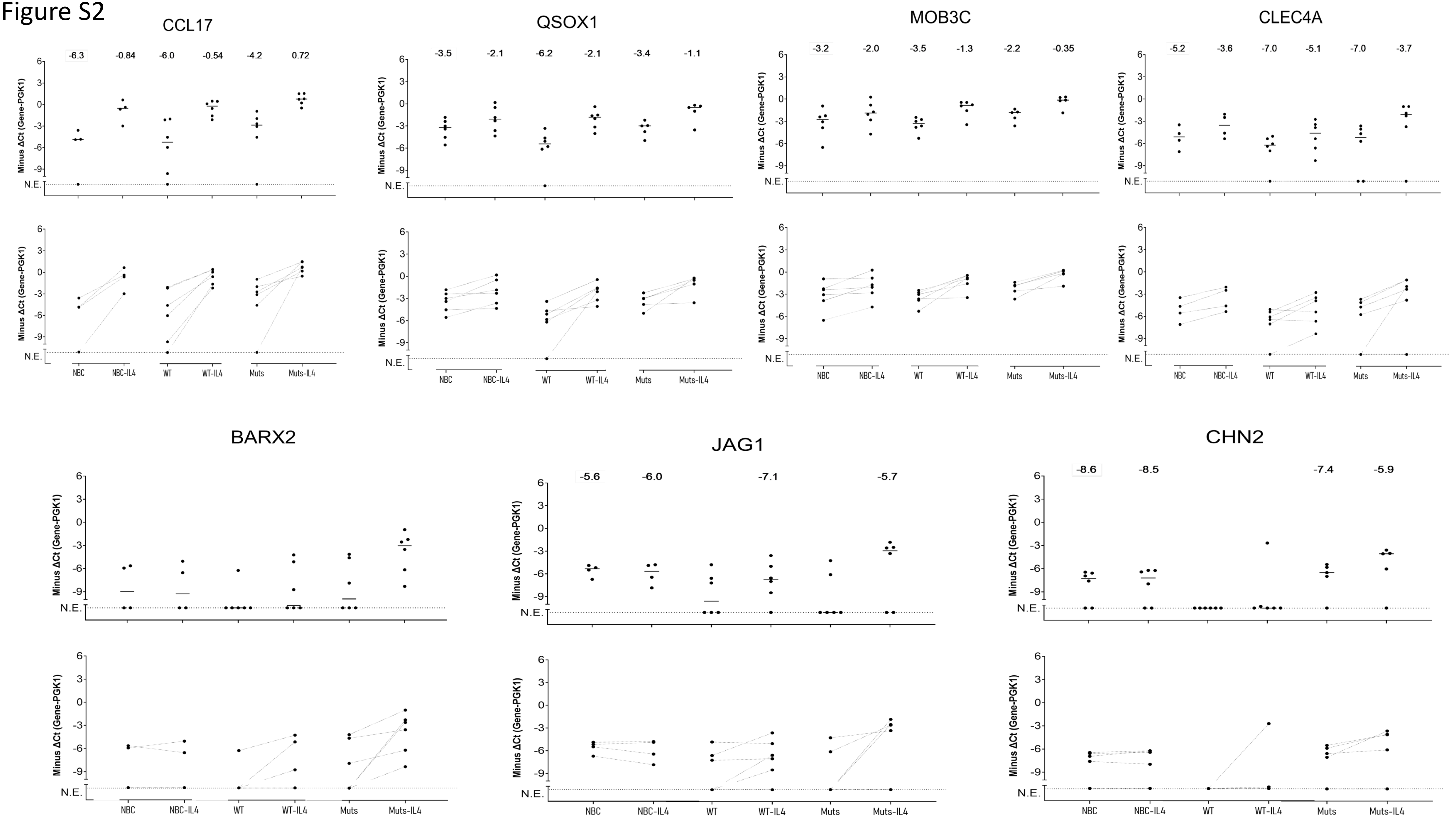
CCL17
QSOX1
MOB3C
CLEC4A
BARX2
JAG1
CHN2
Minus ΔCt (Gene-PGK1)
N.E.
NBC
NBC-IL4
WT
WT-IL4
Muts
Muts-IL4
-6.3 -0.84 -6.0 -0.54 -4.2 0.72
-3.5 -2.1 -6.2 -2.1 -3.4 -1.1
-3.2 -2.0 -3.5 -1.3 -2.2 -0.35
-5.2 -3.6 -7.0 -5.1 -7.0 -3.7
-5.6 -6.0 -7.1 -5.7
-8.6 -8.5 -7.4 -5.9

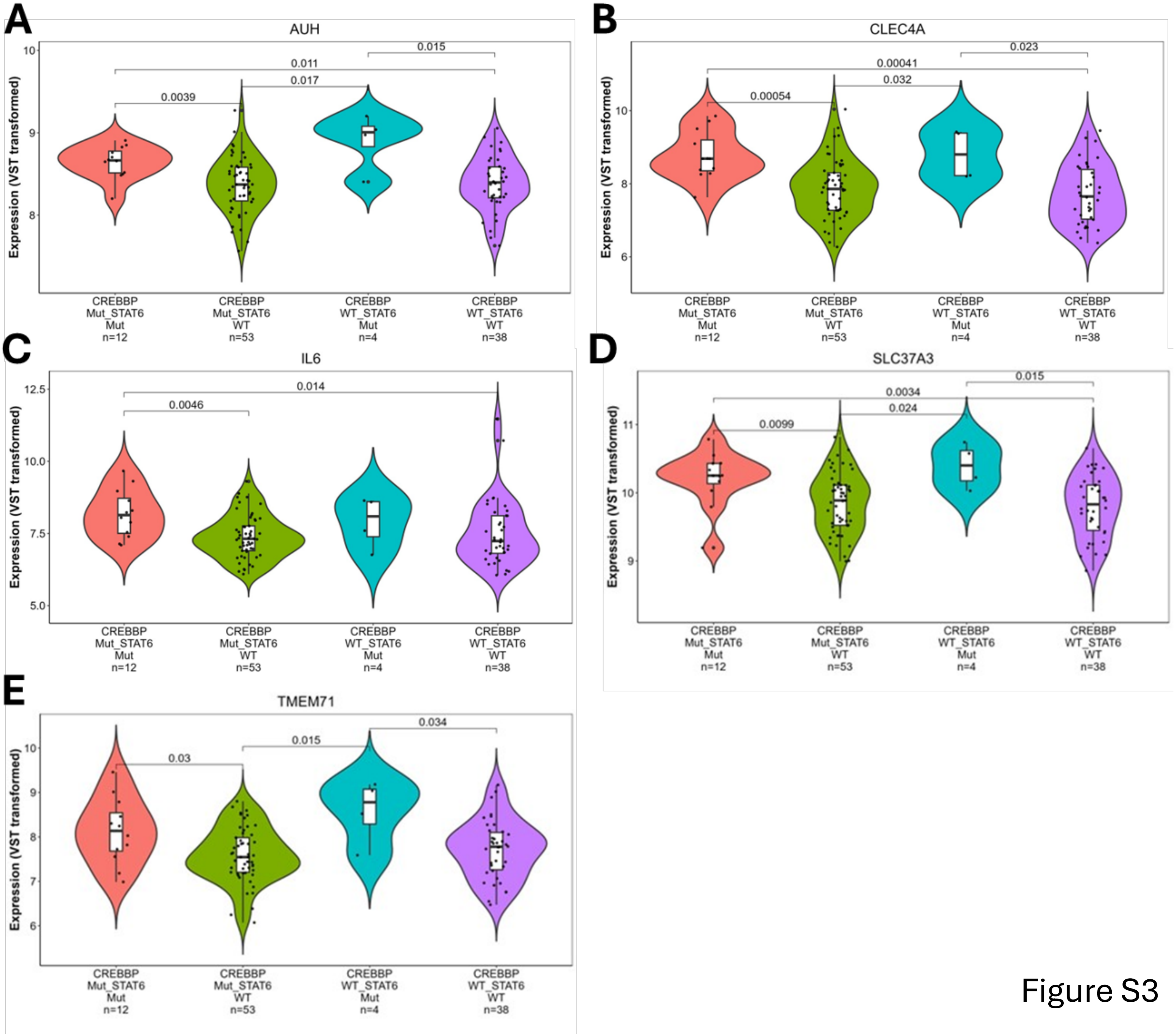
A
AUH
Expression (VST transformed)
10
9
8
0.015
0.011
0.017
0.0039
B
CLEC4A
10
8
6
0.023
0.00041
0.032
0.00054
C
IL6
12.5
10.0
7.5
5.0
0.014
0.0046
D
SLC37A3
11
10
9
0.015
0.0034
0.024
0.0099
E
TMEM71
10
9
8
7
6
0.034
0.015
0.03
CREBBP Mut_STAT6 Mut n=12
CREBBP Mut_STAT6 WT n=53
CREBBP WT_STAT6 Mut n=4
CREBBP WT_STAT6 WT n=38

Figure S3

Figure S4

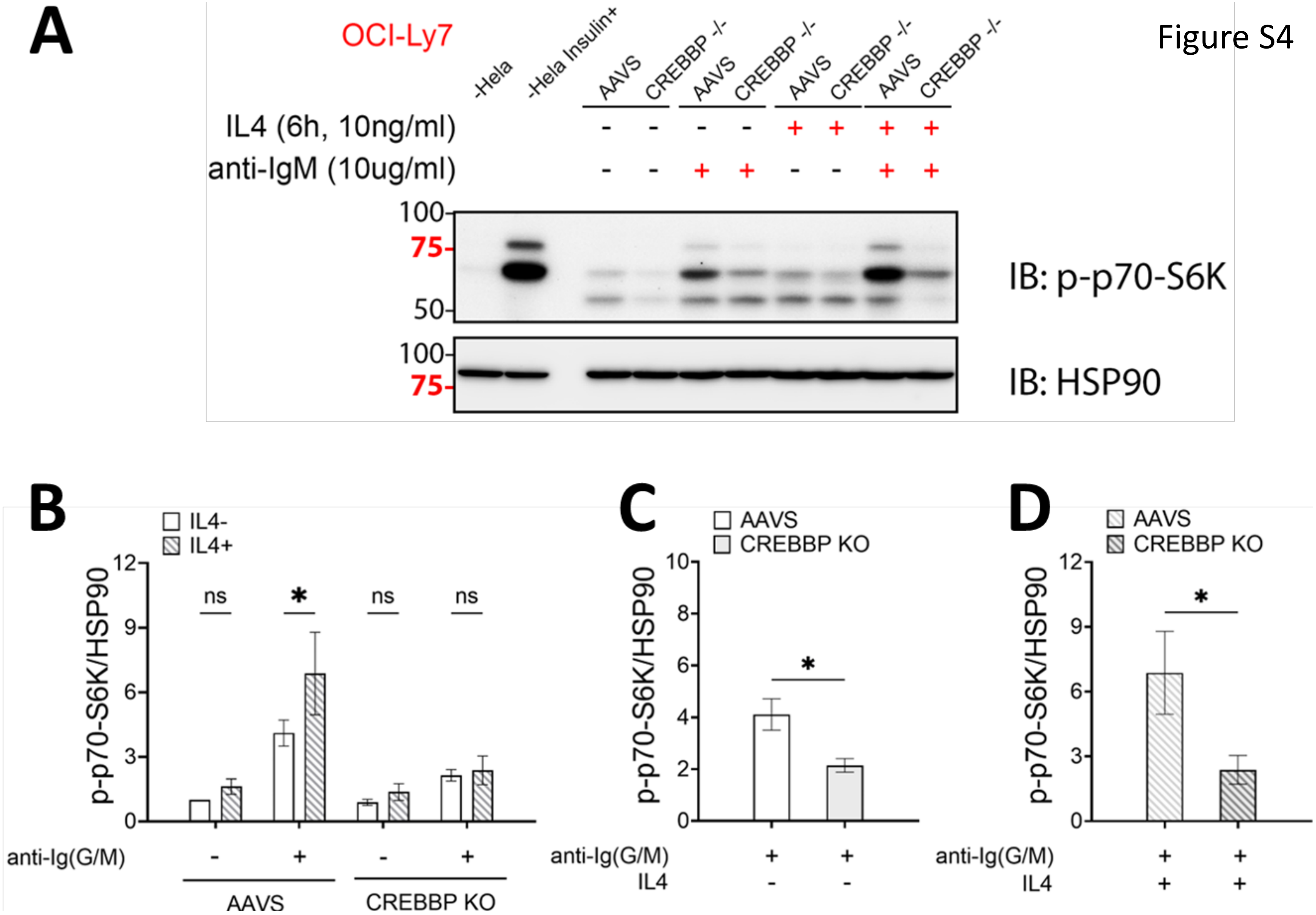
A
OCI-Ly7
-Hela
-Hela Insulin+
AAVS
CREBBP -/-
IL4 (6h, 10ng/ml)
anti-IgM (10ug/ml)
100
75
50
IB: p-p70-S6K
IB: HSP90
B
IL4-
IL4+
p-p70-S6K/HSP90
ns
*
anti-Ig(G/M)
AAVS
CREBBP KO
C
AAVS
CREBBP KO
anti-Ig(G/M)
IL4
D
AAVS
CREBBP KO
anti-Ig(G/M)
IL4
